# Comorbid diseases as risk factors for incident posttraumatic stress disorder (PTSD) in a large community cohort (KCIS no.PSY4)

**DOI:** 10.1038/srep41276

**Published:** 2017-01-27

**Authors:** Jung-Chen Chang, Amy Ming-Fang Yen, Hsiu-Hsi Chen, Sam Li-Sheng Chen, Sherry Yueh-Hsia Chiu, Jean Ching-Yuan Fann, Chau-Shoun Lee

**Affiliations:** 1 School of Nursing, College of Medicine, National Taiwan University, Taipei, Taiwan; National Taiwan University Hospital, Taipei, Taiwan; 2 School of Oral Hygiene, College of Oral Medicine, Taipei Medical University, Taipei, Taiwan; 3 Division of Biostatistics, Graduate Institute of Epidemiology and Preventive Medicine, College of Public Health, National Taiwan University, Taipei, Taiwan; 4 Department of Health Care Management, College of Management, Chang Gung University, Taoyuan, Taiwan; 5 Department of Health Industry Management, College of Healthcare Management, Kainan University, Taoyuan County, Taiwan; 6 Department of Medicine, Mackay Medical College, New Taipei City, Taiwan; 7 Department of Psychiatry, Mackay Memorial Hospital, Taipei, Taiwan

## Abstract

Nature disasters and terrorist attacks have occurred globally in recent years. Posttraumatic stress disorder (PTSD) has gained increasing attention, but its incidence and comorbidities in the general population are different from those inside the disaster areas. The present study estimated incident PTSD and comorbid diseases for over a decade in a cohort from a community-based integrated screening program. Factors associated with the incidence of PTSD were analyzed using Cox regression models. PTSD incidence was estimated as 81 per 10^5^ person-years. Incidence was higher in females than in males and one-year increments in age lowered the risk for PTSD by 3%. Adjusting for other factors, cardiovascular heart disease (adjusted hazard ratio (aHR) = 1.45, 95% confidence interval (CI): 1.03–2.04), bipolar disorder (aHR = 1.86, 95% CI: 1.07–3.24) and major depressive disorder (aHR = 7.03, 95% CI: 5.02–9.85) all significantly increased 45%, 86% and 603%, respectively, the risk of developing PTSD. The low rate of people with incident PTSD receiving treatment in this community health screening population implies there is room for improvement in terms of early detection and intervention. Clinical preventive efforts may be made for patients seeking general medical help, especially those with cardiovascular disorders or mood disorders.

Posttraumatic stress disorder (PTSD) is a common, debilitating mental disorder that describes a syndrome that can occur following traumatic experiences. Lifetime exposure to trauma may reach 50% or higher^1^,^2^. Previously reported 1-year and lifetime prevalence rates of PTSD among adults from general population were around 3.5% and 7%, respectively^3^,^4^,^5^,^6^. However, most previous epidemiological studies have reported the prevalence of PTSD in subjects exposed to war, terrorism, or natural disasters, rather than incidence of the disorder in the general population^7^.

The incidence of PTSD varies across different studies and populations. For example, PTSD incidence rates observed among individuals who survived a cardiac arrest range from 19% to 27%, with comorbid depression and anxiety commonly seen^8^. Among train drivers who had witnessed a railway suicide (a person under the train) the incidence of PTSD ranged from 14% (severe PTSD) to 44% (moderate PTSD)^9^. In survivors of motor vehicle accidents, incidences of PTSD were 7.5% in face-to-face interviews^10^ and 8.5% in telephone interviews^11^.

The incidence of PTSD also varies geographically^2^,^12^. In particular, low prevalence rates (0.2–0.4%) have been observed in Japan and China^13^,^14^,^15^,^16^. The PTSD incidence rate in a Japanese study was much lower than those reported in the United States, United Kingdom, Israel, and Australia, but similar to that in Switzerland^17^,^18^,^19^,^20^,^21^,^22^,^23^. It is possible that discrepancies in the incidence of PTSD between developed and developing countries stem from differences in health care, medical advances, and social norms about caring for people following traumatic events or differences in reporting symptoms or seeking care for PTSD. There have been fewer studies of incidence than prevalence of PTSD, and as such, risk factors associated with incident PTSD have not been well investigated. However, age at traumatic event, female gender, (family) history of psychiatric illness, and lower education level are all reported as pre-traumatic risk factors across trauma types^24^,^25^.

As it is often not feasible to collect data before traumatic events have occurred, investigations of risk factors for incident PTSD are often retrospective rather than prospective^26^,^27^. There is also a wide range of common comorbidities for incident PTSD, including mental illnesses, such as depressive disorders, anxiety, and substance-related disorders, and physical diseases, such as diabetes, obesity, angina, hypertension, gastritis, and arthritis^6^,^28^,^29^,^30^,^31^. However, although antecedent physical conditions, such as metabolic syndrome and cardiovascular disorders, have been shown to be associated with various psychiatric disorders^32^,^33^, these have not been well studied in PTSD.

In this study, we aimed to prospectively investigate mental and physical comorbid diseases associated with incident PTSD in a cohort participating in a community health-screening program. The presence of such potential risk factors is routinely assessed in healthcare settings before the onset of PTSD. If comorbid diseases increase the risk of incident PTSD, enhanced surveillance in high-risk populations may be warranted.

## Methods

### Study participants and procedure

The cohort was derived from a population of about 390 thousands adults (aged 30 years and above) in the northeastern part of Taiwan. The population was invited to participate in a longitudinal community-based study, the Keelung Community-based Integrated Screening (KCIS), between 1999 and 2004. The details of KCIS have been described elsewhere^34^. Briefly, the KCIS program was originally designed as a continuing evidence-based screening program for five neoplastic and three non-neoplastic diseases (type 2 diabetes, hypertension, and hyperlipidemia) with inter-screening intervals ranging from 1 to 5 years (mean: 3.13, SD: 1.47). As the KCIS program is a multiple screening program, the follow-up of this cohort enabled us to ascertain multiple outcomes on neoplastic diseases and other chronic disorders, such as mental illnesses (including PTSD).

We excluded participants with a current or previous diagnosis of PTSD at baseline. Individuals at-risk were followed up from 1999 to 2004 to calculate the incidence of PTSD. In this prospective design, each participant entered the study at some point between 1999 and 2004, and the follow-up period depended on the entrance date. The coverage rate (percentage of participation) of residents enrolled into the KCIS program was approximately 55% in 2004^35^. As a result, there were approximately 138,420 target population eligible for inclusion, yielding 76,545 at-risk participants for the PTSD incidence survey. Of these, 76,417 completed data collection for the final analysis. The exclusion of 128 subjects was due to missing data on essential variables, such as those about clinical diagnoses, metabolic components, and life style details.

Community nurses administrated questionnaires, which inquired about social demographics and life style details (e.g., smoking or alcohol drinking habits etc.) that might be associated with PTSD. Participants were not aware of any hypothesized associations between the risk factors and PTSD before screening. The study hypothesis was formulated independent from PTSD and comorbid aliments ascertainment.

### Data Collection

On entering the program, enrolled participants were interviewed to collect social demographics, health behaviors (e.g., smoking or alcohol drinking), and family history of major illnesses (e.g., psychiatric disorders). Assessment of smoking and alcohol consumption was dichotomous: current use or no current use. Further examinations included reclining blood pressure (BP) after 5 to 10 minutes of rest and a fasting blood sample to assess glucose, triglyceride (TG), and high-density lipoprotein (HDL) cholesterol levels. Trained staff measured participants’ heights using a stadiometer, waist circumference (WC, to the nearest 0.1 cm) by a standard tape, and body weight (to the nearest 0.1 kg) by a standardized weight scale. The waist size was checked horizontally at the midway between the inferior margin of the rib cage and the iliac crest.

### Measurement of metabolic syndrome (MetS) and its components

MetS was diagnosed according to the NCEP ATP III criteria^36^ with the adjustment for waist size in Asian subjects^37^. We defined participants as having MetS if three or more of the following criteria were met: (1) central obesity (an increased WC, WC ≥80 cm for women and ≥90 cm for men); (2) high TG (TG ≥ 150 mg/dL); (3) a low HDL cholesterol concentration (<50 mg/dL for women and <40 mg/dL for men); (4) an elevated BP (≥130 mmHg systolic or ≥85 mmHg diastolic); and (5) glucose intolerance (elevated fasting glucose ≥110 mg/dL).

### Diagnosis of PTSD

Data used for identifying patients with PTSD were drawn from a national population-based dataset, the National Health Insurance Dataset (NHID). In 1995, a government supported single-payer national health care system was implemented in Taiwan. This system offers affordable and rapid medical attention. National Health Insurance, one of the most complete health insurance systems in the world, finances healthcare for almost all (>99%) 23 million Taiwanese citizens. The NHID provides details on medical visits, including International Classification of Diseases (ICD) codes, dates of visits, and so on. This universal NHID offers a comparable prognosis with an affordable premium relative to other large series of health insurance datasets in developed countries. In the present study, individuals in the NHID were diagnosed with PTSD according to the ICD-9 (codes 309 s), ascertained by psychiatrists’ or physicians’ clinical diagnosis.

### Information on comorbid medical and mental disorders

To assess diseases that are comorbid with PTSD, we retrieved data on several major illnesses from the NHID, which diagnoses were ascertained by psychiatrist or physician in each specialty. This included neoplasm (ICD codes 140–208), diabetes mellitus (DM, 250), hypertensive disorders (HTN, 401–405), cardiovascular diseases (CVD, 390–398, 410–414, and 420–429), and cerebrovascular accidents (CVA, 430–438). Mood-related psychiatric disorders, relevant to the development of PTSD, were enrolled for this investigation. We defined major depressive disorders (MDD) according to the ICD diagnostic codes 296.20–296.26, 296.30–296.36, and 296.82. Bipolar disorders (BPD) were defined according to the codes 296.00 to 296.06, 296.10 to 296.16, 296.40 to 296.46, 296.50 to 296.56, 296.60 to 296.66, 296.7, 296.80, 296.81, 296.89, and 296.90). Anxiety disorders were defined according to the codes 300.0x. Substance use disorders (SUD) were defined according to the codes 291 s, 292 s, 303 s, 304 s, and 305 s. The comorbid medical and mental disorders were clinically diagnosed by a psychiatrist or physician based on ICD or DSM (Diagnostic and Statistical Manual of Mental disorders) criteria. Information on medical history was obtained from the NHID. To ensure the validity of diagnoses, diseases were identified according to the respective ICD-9 codes at least three times outpatient visits or one hospital admission.

### Statistical analyses

For descriptive analyses, continuous variables were expressed as means (±SD) and categorical variables were expressed as percentages. To calculate the incidence of PTSD, person-years at risk since the date of attending the first screening were calculated. Eligible participants ceased to contribute to person-years at census either after their final recorded assessment or when they met the criteria for PTSD.

We first used univariate Cox proportional hazards regression models to estimate the hazard ratio of developing PTSD with demographic and clinical characteristics as covariates. A multivariable Cox regression model was further conducted with a stepwise selection method set at 0.10 for inclusion or exclusion of variables within each criterion. Finally, two-tailed tests with α = 0.05 were considered statistically significant. All statistical analyses were carried out using SAS software (version 9.2; SAS Institute Inc, Cary, NC).

### Ethical consideration and funding source

We used data derived from a large research project (KCIS)^34^. The original research protocol was reviewed and approved by the ethical review committee of National Taiwan University. The KCIS program performs annual recruitment screenings, which are approved by the local ethical committee in the Health Bureau of Keelung City. These approvals include data linkage systems and strict maintenance of participant confidentiality. All methods were performed in accordance with the relevant guidelines and regulations.

Written informed consent was obtained from each participant at the time of recruitment to the program^34^. Because the personal identification numbers for the datasets were encoded, the privacy and confidentiality of patients were ensured by obscuring the links between datasets. None of the authors received outside funding for the current study.

## Results

### Cumulative incidence of PTSD

**Figure 1:**
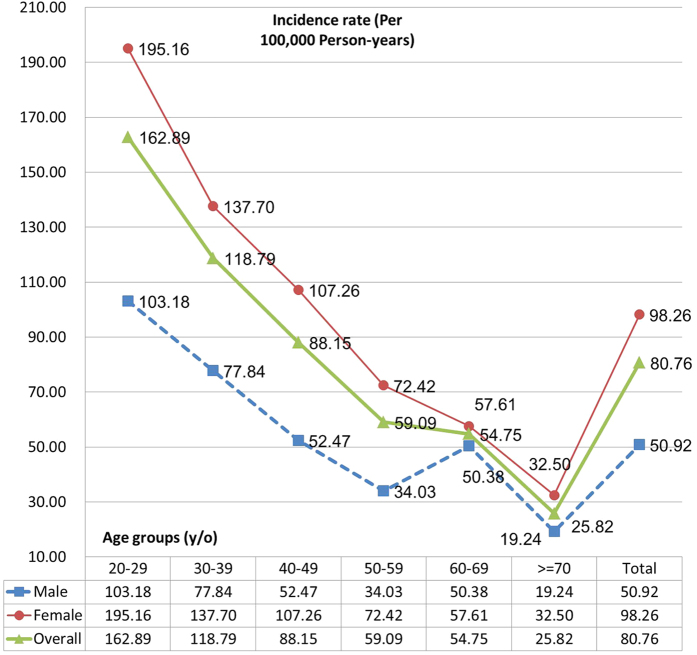
Age- and gender-specific incidence of PTSD.

Among 76,417 at-risk subjects followed until the end of 2004, 193 incident PTSD cases were found. Of these, 148 were female (76.7%). The average age of PTSD cases was 43.5 (S.D. 13.8). The overall incident rate of PTSD was 0.08% (95% CI = 0.06–0.10%). Figure 1 shows age- and gender-specific incidences of PTSD. Incidence of PTSD was higher for female than male participants across all age groups. Subjects aged 20 to 29 years had the highest incidence of PTSD for both genders (detailed statistics are shown in Table 1).

In the univariate logistic regression, compared with non-PTSD participants, those with PTSD tended to be female, younger, more highly educated, non-smokers, and non-drinkers. Furthermore, participants with PTSD had a higher proportion of CVD, MDD, anxiety disorders, BPD, and SUD. MetS, however, demonstrated a minor protective effect for the occurrence of PTSD (Table 2).

### Risk factors for developing PTSD at follow-up

Table 3 shows the results of the univariate Cox proportional hazard regression models, indicating that male gender and older age were significantly inversely associated with PTSD incidence. Therefore, we subsequently controlled for the effects of sex and gender, and found that the onset of PTSD was significantly associated with low education, physical illnesses (cancer, diabetes, hypertension, CVA, and CVD), and mental disorders (MDD, anxiety disorders, BPD, and SUD).

The multivariable Cox regression analysis showed that MDD accounted for around a 7-fold increase in risk for developing PTSD (aHR = 7.03, 95% CI = 5.02–9.85). Bipolar disorders were also associated with the incident PTSD (aHR = 1.86, 95% CI = 1.07–3.24). CVD increased incident PTSD risk by 45% (95% CI = 1.03–2.04). One-year increments in age lowered the risk of PTSD by 3% (aHR = 0.97, 95% CI = 0.95–0.98). Education level was significantly associated with PTSD, with those educated to elementary school or lower having half the risk of PTSD compared with those educated to college or above (aHR = 0.54, 95% CI = 0.32–0.90).

## Discussion

The strengths of this study include the large sample size from a general population, long follow-up period, and linked data from multiple sources. In this large community cohort, we found an overall incident rate of PTSD as low as 0.08%. However, various physical and psychological antecedents were common in the development of PTSD. In our multivariable analyses, MDD accounted for a greater than 6-fold increase in hazard ratio for PTSD and BPD and CVD increased the hazard ratio by around 86% and 45%, respectively. MetS was not found to be associated with the development of PTSD after adjustment for covariates.

We identified incident cases among those receiving clinical diagnoses and healthcare services. The cumulative incidence rate in our study is relatively low, but is consistent with rates of PTSD after a traumatic experience reported in another Eastern society^15^,^16^ and in Asian Americans^38^. In a survey of common psychiatric disorders among a nationally representative sample of Taiwanese, similarly low rates of MDD were also reported^39^. One possible explanation for this is the low level of help-seeking behavior for mental disorders commonly seen in Asian countries^40^ and the limited understanding of PTSD in the general public. When PTSD affects US ethnic minorities, the mental illness is usually undertreated, especially in Asian groups^38^. This indicates room for improvement in the detection of PTSD in health screening programs in Asian countries^41^. Alternatively, under-diagnosis may also arise from the insufficient awareness of PTSD in clinicians^39^,^41^. In a recent World Mental Health (WMH) Surveys in 18 countries, disaster-related PTSD prevalence was only 0.0–3.8% and was significantly related to high education and other risk factors^42^. The present study also found low rate of PTSD and related to higher educated persons. It may be explained by high level of awareness of PTSD and willingness to seek help from medical professionals in higher than lower educated persons.

Associations between CVD and PTSD have been consistently reported in the general population^43^. Possible mechanisms for this link include cytokines, increased allostatic load, the hypothalamic–pituitary–adrenal axis, the autonomic nervous system, psychiatric risk factors (e.g., substance abuse, depression), shared genetic vulnerability, and MetS^44^,^45^,^46^,^47^,^48^,^49^. However, most studies have investigated the impact of PTSD on the risk or outcome of CVD^50^. In the present study, we investigated the relationship in the opposite direction to find that CVD played a role in the development of PTSD. Recent evidence has also highlighted the bidirectional relationship between PTSD and CVD^44^. Life-threatening medical illness can elicit PTSD, and conversely, PTSD increases subsequent CVD events and mortalities. A common physical basis for CVD and PTSD, particularly hyperarousal symptoms, is sympathetic activation, as well as higher levels of coronary artery calcium^51^. Another possible pathway between cardiovascular events and PTSD is medication nonadherence causing exacerbated physiological conditions and an unhealthy lifestyle, such as sleep disturbances^52^.

The co-occurrence of PTSD and depressive disorders is common^53^,^54^. However, the relational direction and nature of this association is unclear. It has been suggested that these phenomena have overlapping symptoms with similar trait vulnerability. In our longitudinal study, MDD was found to predict the onset of PTSD. Having comorbid depression was one of the major risk factors for newly diagnosed PTSD among women who have been sexually assaulted^55^. Pre-existing depression was a consistent independent risk factor for intensive care unit- related PTSD at both 3 and 12 months^56^. A lack of awareness of PTSD complaints is common and post-traumatic suffering is often silent, because other comorbid disorders and complaints are more prominent early in the clinical picture following traumatic experiences. Thus a depressive episode characterized as help-seeking signal or drug-pursuing behavior is frequently encountered^57^. Our findings could also partially be explained by a lack of awareness among patients and clinicians. Moreover, the knowledge, skills and ability to diagnose depressive disorders are more sophisticated than for PTSD in general physicians and public health workers. More people with depressive disorders are therefore likely to be detected and managed than those with PTSD^58^. Identifying those at the greatest risk of developing PTSD can allow adequate allocation of therapeutic resources. Finally, we also found the correlation between BPD and PTSD. The childhood traumatic experiences have been shown to associate with BPD^59^. It might explain why the patient with BPD was vulnerable to the subsequent development of PTSD in this study.

### Limitations

It is worth considering a few limitations in the current study. First, some further factors related to the development of PTSD might not have been considered in our study, such as previous experience of trauma and personality traits. Furthermore, the later PTSD diagnosis could not be differentiated as the same or new course with the prior one. Therefore, previous history of PTSD was not included as a risk factor for incident case.

Second, the data used in the current study are limited to subjects attending the community-based integrated screening program. Whether PTSD cases identified in the screened cohort are generalizable to those in a non-screened cohort is unclear.

Third, PTSD cases had to have been treated or diagnosed in a clinical setting in order to be in the NHID. This study likely underestimated the true number of PTSD cases, as many people might not seek care and go undiagnosed.

### Clinical Implications

Our findings are consistent with the concept that subjects with CVD or affective disorders (both unipolar and bipolar disorders) may represent a high risk group for developing PTSD in the community. Traumatized people are far more likely to visit primary care physicians than mental health professionals to seek help for physical discomfort and mental symptoms. Therefore, general practitioners will play an important role in identifying and treating PTSD^41^,^60^.

## Additional Information

**How to cite this article**: Chang, J.-C. *et al*. Comorbid diseases as risk factors for incident posttraumatic stress disorder (PTSD) in a large community cohort (KCIS no.PSY4). *Sci. Rep.* **7**, 41276; doi: 10.1038/srep41276 (2017).

**Publisher's note:** Springer Nature remains neutral with regard to jurisdictional claims in published maps and institutional affiliations.

## Figures and Tables

**Table 1:** Age- and gender-specific incidence of PTSD in a large community longitudinal cohort (n = 76,417).

Age (years)	Male			Female			Both genders		
	Cases	PY	Incidence (×10^−5^)	Cases	PY	Incidence (×10^−5^)	Cases	PY	Incidence (×10^−5^)
20–29	6	5815	103.18	21	10760	195.16	27	16576	162.89
30–39	12	15417	77.84	46	33407	137.70	58	48824	118.79
40–49	11	20966	52.47	42	39158	107.26	53	60124	88.15
50–59	5	14692	34.03	20	27615	72.42	25	42307	59.09
60–69	8	15880	50.38	14	24299	57.61	22	40179	54.75
>=70	3	15596	19.24	5	15385	32.50	8	30981	25.82
Total	45	88367	50.92	148	150625	98.26	193	238991	80.76

PY = person-years.

**Table 2:** Demographic and clinical characteristics by the status of PTSD in the univariate logistic regression.

Variables		With PTSD		Without PTSD		p-value
Gender	Male	45	0.15%	29180	99.85%	<0.0001
	Female	148	0.31%	47044	99.69%	
Age (Mean (SD))	years	43.5 (13.8)		49.6 (15.3)		<0.0001
Education	Low	45	0.15%	29169	99.85%	<0.0001
	Medium	95	0.29%	32522	99.71%	
	High	53	0.36%	14492	99.64%	
Marriage	Single	26	0.24%	10682	99.76%	0.2545
	Married	138	0.24%	56915	99.76%	
	Widow	29	0.34%	8574	99.66%	
Smoking	No	157	0.29%	54075	99.71%	0.0006
	Yes	31	0.15%	20801	99.85%	
Drinking	No	163	0.28%	57105	99.72%	0.0007
	Yes	24	0.14%	17410	99.86%	
Cancer	No	173	0.25%	70330	99.75%	0.172
	Yes	20	0.34%	5894	99.66%	
Diabetes	No	149	0.25%	59532	99.75%	0.7629
	Yes	44	0.26%	16692	99.74%	
Hypertension	No	130	0.26%	49864	99.74%	0.5715
	Yes	63	0.24%	26360	99.76%	
CVA	No	170	0.25%	68310	99.75%	0.4852
	Yes	23	0.29%	7914	99.71%	
CVD	No	122	0.23%	53589	99.77%	0.0313
	Yes	71	0.31%	22635	99.69%	
MetS	No	174	0.28%	62434	99.72%	0.0022
	Yes	18	0.13%	13543	99.87%	
MDD	No	130	0.18%	72117	99.82%	<0.0001
	Yes	63	1.51%	4107	98.49%	
ANX	No	182	0.24%	74686	99.76%	0.0003
	Yes	11	0.71%	1538	99.29%	
SUD	No	187	0.25%	75510	99.75%	0.0018
	Yes	6	0.83%	714	99.17%	
BPD	No	170	0.23%	74745	99.77%	<0.0001
	Yes	16	1.07%	1479	98.93%	

1 Education: Elementary schools or below (low); High school (medium); University (high). 2 CVA: cerebrovascular accidents; CVD: cardiovascular diseases; MetS: metabolic syndrome; MDD: major depressive disorders; ANX: anxiety disorders; SUD: substances use disorders; BPD: bipolar disorders.

**Table 3:** Estimated hazard ratios of demographic and clinical characteristics for incident PTSD by proportional hazards regression model.

Variables		HR*	95%	CI	p-value	aHR	95%	CI	p-value
**Gender**	**Male**	**0.57**	**0.41**	**0.79**	**0.0009**	**0.70**	**0.47**	**1.05**	**0.0805**
	**Female**	**1.00**				**1.00**			
**Age (Mean (SD)**	**year**	**0.97**	**0.96**	**0.98**	**<0.0001**	**0.97**	**0.95**	**0.98**	**<0.0001**
**Education**	**Low**	**0.48**	**0.29**	**0.79**	**0.0152**	**0.54**	**0.32**	**0.90**	**0.0641**
	**Medium**	**0.75**	**0.53**	**1.06**		**0.78**	**0.54**	**1.12**	
	**High**	**1.00**			**1.00**				
Marriage	Single	1.31	0.84	2.04	0.4690				
	Married	1.00							
	Widow	1.07	0.69	1.66					
Smoking	No	1.00			0.1042	1.00			0.0513
	Yes	0.69	0.45	1.08		0.64	0.40	1.00	
Drinking	No	1.00			0.0500				
	Yes	0.63	0.39	1.00					
Cancer	No	1.00			0.0140	1.00			0.0675
	Yes	1.81	1.13	2.90		1.57	0.97	2.52	
Diabetes	No	1.00			0.0239				
	Yes	1.52	1.06	2.18					
Hypertension	No	1.00			0.0046				
	Yes	1.68	1.18	2.42					
CVA	No	1.00			0.0028				
	Yes	2.06	1.28	3.30					
**CVD**	**No**	**1.00**			**<0.0001**	**1.00**			**0.0345**
	**Yes**	**2.14**	**1.56**	**2.96**		**1.45**	**1.03**	**2.04**	
MetS	No	1.00			0.5024				
	Yes	0.84	0.51	1.39					
**MDD**	**No**	**1.00**			**<0.0001**	**1.00**			**<0.0001**
	**Yes**	**8.57**	**6.34**	**11.60**		**7.03**	**5.02**	**9.85**	
ANX	No	1.00			0.0004				
	Yes	3.04	1.65	5.60					
SUD	No	1.00			0.0011				
	Yes	3.91	1.73	8.84					
BPD	No	**1.00**			**<0**.**0001**	1.00			**0.0291**
	Yes	10.08	7.55	13.46		1.86	1.07	3.24	

*1 Hazard ratios of education, marriage, smoking, drinking, physical illnesses, and mental disorders were adjusted by age and gender. 2 Education: Elementary schools or below (low); High school (medium); University (high). 3 HR, hazard ratio; aHR, adjusted hazard ratio. 4 CVA: cerebrovascular accidents; CVD: cardiovascular diseases; MetS: metabolic syndrome; SUD: substances use disorders; MDD: major depressive disorders; ANX: anxiety disorders; BPD: bipolar disorders.

## References

[b1] Breslau N. . Trauma and posttraumatic stress disorder in the community: the 1996 Detroit Area Survey of Trauma. Arch Gen Psychiatry. 55, 626–632 (1998). 9672053 10.1001/archpsyc.55.7.626

[b2] U.S. Department of Veterans Affairs. *How common is PTSD? (2015)*. Retrieved 21 March 2016 from Available at: http://www.ptsd.va.gov/public/PTSD-overview/basics/how-common-is-ptsd.asp (Accessed: 21th March 2016).

[b3] Kessler R. C. . Lifetime prevalence and age-of-onset distributions of DSM-IV disorders in the National Comorbidity Survey Replication. Arch Gen Psychiatry. 62, 593–602 (2005). 15939837 10.1001/archpsyc.62.6.593

[b4] Kessler R. C., Chiu W. T., Demler O., Merikangas K. R. & Walters E. E. Prevalence, severity, and comorbidity of 12-month DSM-IV disorders in the National Comorbidity Survey Replication. Arch Gen Psychiatry. 62, 617–627 (2005). 15939839 10.1001/archpsyc.62.6.617 PMC2847357

[b5] Bromet E., Sonnega A. & Kessler R. C. Risk factors for DSM-III-R posttraumatic stress disorder: findings from the National Comorbidity Survey. Am J Epidemiol. 147, 353–361 (1998). 9508102 10.1093/oxfordjournals.aje.a009457

[b6] Pietrzak R. H., Goldstein R. B., Southwick S. M. & Grant B. F. Medical comorbidity of full and partial posttraumatic stress disorder in US adults: results from Wave 2 of the National Epidemiologic Survey on Alcohol and Related Conditions. Psychosom Med. 73, 697–707 (2011). 21949429 10.1097/PSY.0b013e3182303775 PMC3188699

[b7] Kessler R. C., Sonnega A., Bromet E., Hughes M. & Nelson C. B. Posttraumatic stress disorder in the National Comorbidity Survey. Arch Gen Psychiatry. 52, 1048–60 (1995). 7492257 10.1001/archpsyc.1995.03950240066012

[b8] Wilder Schaaf K. P. . Anxiety, depression, and PTSD following cardiac arrest: a systematic review of the literature. Resuscitation. 84, 873–877 (2013). 23200996 10.1016/j.resuscitation.2012.11.021

[b9] Mehnert A., Nanninga I., Fauth M. & Schäfer I. Course and predictors of posttraumatic stress among male train drivers after the experience of ‘person under the train’ incidents. J Psychosom Res. 73, 191–196 (2012). 22850259 10.1016/j.jpsychores.2012.06.007

[b10] Nishi D. . Incidence and prediction of post-traumatic stress disorder at 6 months after motor vehicle accidents in Japan. Psychosomatics. 54, 263–271 (2013). 23194933 10.1016/j.psym.2012.07.010

[b11] Hamanaka S. . Acute stress disorder and posttraumatic stress disorder symptoms among patients severely injured in motor vehicle accidents in Japan. Gen Hosp Psychiatry. 28, 234–241 (2006). 16675367 10.1016/j.genhosppsych.2006.02.007

[b12] Tol W. A., Barbui C. & van Ommeren M. Management of acute stress, PTSD, and bereavement: WHO recommendations. JAMA. 310, 477–478 (2013). 23925613 10.1001/jama.2013.166723

[b13] Bromet E. J. & Gluzman S. F. The state of mental health and alcoholism in Ukraine. In The WHO World Mental Health Surveys (ed. Ronald T. B. U. & Kessler C.) pp. 431–445 Geneva Switzerland: Cambridge (2008).

[b14] Graaf Ron de. & Ormel J. Mental disorders and service use in the Netherlands: Results from the European Study of the Epidemiology of Mental Disorders (ESEMeD). In The WHO World Mental Health Surveys (ed. Ronald T. B. U. & Kessler C.) pp. 388–405 Geneva, Switzerland: Cambridge (2008).

[b15] Huang Y., Liu Z. & Zhang M. Mental disorders and service use in China. In The WHO World Mental Health Surveys (ed. Ronald T. B. U. & Kessler C.) pp. 447–473 Geneva, Switzerland: Cambridge (2008).

[b16] Kawakami N. & Takeshima T. Twelve-month prevalence, severity, and treatment of common mental disorders in communities in Japan. In The WHO World Mental Health Surveys (ed. Ronald T. B. U. & Kessler C.) pp. 474–485 Geneva Switzerland: Cambridge (2008).

[b17] Bryant R. A., Creamer M., O′Donnell M., Silove D. & McFarlane A. C. A multisite study of initial respiration rate and heart rate as predictors of posttraumatic stress disorder. J Clin Psychiatry. 69, 1694–1701 (2008). 19014750 10.4088/jcp.v69n1104

[b18] Mayou R., Bryant B. & Ehlers A. Prediction of psychological outcomes one year after a motor vehicle accident. Am J Psychiatry. 158, 1231–1238 (2001). 11481156 10.1176/appi.ajp.158.8.1231

[b19] O’Donnell M. L., Creamer M., Pattison P. & Atkin C. Psychiatric morbidity following injury. Am J Psychiatry. 161, 507–514 (2004). 14992977 10.1176/appi.ajp.161.3.507

[b20] Schnyder U., Moergeli H., Klaghofer R. & Buddeberg C. Incidence and prediction of posttraumatic stress disorder symptoms in severely injured accident victims. Am J Psychiatry. 158, 594–599 (2001). 11282694 10.1176/appi.ajp.158.4.594

[b21] Schnyder U., Wittmann L., Friedrich-Perez J., Hepp U. & Moergeli H. Posttraumatic stress disorder following accidental injury: rule or exception in Switzerland? Psychother Psychosom. 77, 111–118 (2008). 18230944 10.1159/000112888

[b22] Shalev A. Y. . Prospective study of posttraumatic stress disorder and depression following trauma. Am J Psychiatry. 155, 630–637 (1998). 9585714 10.1176/ajp.155.5.630

[b23] Zatzick D. F. . A nationwide US study of post-traumatic stress after hospitalization for physical injury. Psychol Med. 37, 1469–1480 (2007). 17559704 10.1017/S0033291707000943

[b24] Brewin C. R., Andrews B. & Valentine J. D. Meta-analysis of risk factors for posttraumatic stress disorder in trauma-exposed adults. J Consult Clin Psychol. 68, 748–766 (2000). 11068961 10.1037//0022-006x.68.5.748

[b25] Ozer E. J., Best S. R., Lipsey T. L. & Weiss D. S. Predictors of posttraumatic stress disorder and symptoms in adults: a meta-analysis. Psychol Bull. 129, 52–73 (2003). 12555794 10.1037/0033-2909.129.1.52

[b26] Koopman C., Classen C. & Spiegel D. Predictors of posttraumatic stress symptoms among survivors of the Oakland/Berkeley, Calif., firestorm. Am J Psychiatry. 151, 888–894 (1994). 8184999 10.1176/ajp.151.6.888

[b27] Widom C. S. Posttraumatic stress disorder in abused and neglected children grown up. Am J Psychiatry. 156, 1223–1229 (1999). 10450264 10.1176/ajp.156.8.1223

[b28] Creamer M., Burgess P. & McFarlane A. C. Post-traumatic stress disorder: findings from the Australian National Survey of Mental Health and Well-being. Psychol Med. 31, 1237–1247 (2001). 11681550 10.1017/s0033291701004287

[b29] Breslau N. Epidemiologic studies of trauma, posttraumatic stress disorder, and other psychiatric disorders. Can J Psychiatry. 47, 923–929 (2002). 12553127 10.1177/070674370204701003

[b30] Kibler J. L., Joshi K. & Ma M. Hypertension in relation to posttraumatic stress disorder and depression in the US National Comorbidity Survey. Behav Med. 34, 125–132 (2009). 19064371 10.3200/BMED.34.4.125-132

[b31] Vieweg W. V. . Posttraumatic stress disorder in male military veterans with comorbid overweight and obesity: psychotropic, antihypertensive, and metabolic medications. Prim Care Companion J Clin Psychiatry. 8, 25–31 (2006). 16862250 10.4088/pcc.v08n0104 PMC1510907

[b32] Chang J. C. . A population-based cohort study to elucidate temporal relationship between schizophrenia and metabolic syndrome (KCIS no. PSY3). Schizophr Res. 151, 158–164 (2013). 24103573 10.1016/j.schres.2013.09.017

[b33] Chang J. C. . Metabolic syndrome and the risk of suicide: a community-based integrated screening samples cohort study. Psychosom Med. 75, 807–814 (2013). 24163389 10.1097/PSY.0000000000000014

[b34] Chen T. H. . Community-based multiple screening model: design, implementation, and analysis of 42,387 participants. Cancer. 100, 1734–1743 (2004). 15073864 10.1002/cncr.20171

[b35] Wang P. E., Wang T. T., Chiu Y. H., Yen A. M. & Chen T. H. Evolution of multiple disease screening in Keelung: a model for community involvement in health interventions? J Med Screen. 13 (Suppl. 1), S54–S58 (2006). 17227644

[b36] Expert Panel on Detection Evaluation and Treatment of High Blood Cholesterol In Adults. Executive Summary of The Third Report of The National Cholesterol Education Program (NCEP) Expert Panel on Detection, Evaluation, And Treatment of High Blood Cholesterol In Adults (Adult Treatment Panel III). JAMA. 285, 2486–2497 (2001). 11368702 10.1001/jama.285.19.2486

[b37] World Health Organization. The Asia-Pacific perspective: redefining obesity and its treatment. WHO: Geneva (2000).

[b38] Roberts A. L., Gilman S. E., Breslau J., Breslau N. & Koenen K. C. Race/ethnic differences in exposure to traumatic events, development of post-traumatic stress disorder, and treatment-seeking for post-traumatic stress disorder in the United States. Psychol Med. 41, 71–83 (2011). 20346193 10.1017/S0033291710000401 PMC3097040

[b39] Liao S. C. . Low prevalence of major depressive disorder in Taiwanese adults: possible explanations and implications. Psychol Med. 42, 1227–1237 (2012). 22051196 10.1017/S0033291711002364

[b40] Chen S. L. S. . A 10-year follow-up study on suicidal mortality after 1999 Taiwan Earthquake. J Psychiatr Res. 79, 42–49 (2016). 27155808 10.1016/j.jpsychires.2016.04.007

[b41] Greene T., Neria Y. & Gross R. Prevalence, detection and correlates of PTSD in the primary care setting: A systematic review. J Clin Psychol Med Settings. 23, 160–80 (2016). 26868222 10.1007/s10880-016-9449-8

[b42] Bromet E. J. . Post-traumatic stress disorder associated with natural and human-made disasters in the World Mental Health Surveys. Psychol Med. 30, 1–15 (2016). 10.1017/S0033291716002026 PMC5432967 27573281

[b43] Spitzer C. . Trauma, posttraumatic stress disorder, and physical illness: findings from the general population. Psychosom Med. 71, 1012–1017 (2009). 19834051 10.1097/PSY.0b013e3181bc76b5

[b44] Edmondson D. & Cohen B. E. Posttraumatic stress disorder and cardiovascular disease. Prog Cardiovasc Dis. 55, 548–556 (2013). 23621964 10.1016/j.pcad.2013.03.004 PMC3639489

[b45] Coughlin S. S. Post-traumatic stress disorder and cardiovascular disease. Open Cardiovasc Med J. 5, 164–170 (2011). 21792377 10.2174/1874192401105010164 PMC3141329

[b46] Boscarino J. A. Post-traumatic stress disorder and cardiovascular disease link: time to identify specific pathways and interventions. Am J Cardiol. 108, 1052–1053 (2011). 10.1016/j.amjcard.2011.07.003 21920186

[b47] Wentworth B. A. . Post-traumatic stress disorder: a fast track to premature cardiovascular disease? Cardiol Rev. 21, 16–22 (2013). 22717656 10.1097/CRD.0b013e318265343b

[b48] Bedi U. S. & Arora R. Cardiovascular manifestations of posttraumatic stress disorder. J Natl Med Assoc. 99, 642–649 (2007). 17595933 PMC2574374

[b49] Dedert E. A., Calhoun P. S., Watkins L. L., Sherwood A. & Beckham J. C. Posttraumatic stress disorder, cardiovascular and metabolic disease: a review of the evidence. Ann Behav Med. 39, 61–78 (2010). 20174903 10.1007/s12160-010-9165-9 PMC3219414

[b50] Jordan H. T., Miller-Archie S. A., Cone J. E., Morabia A. & Stellman S. D. Heart disease among adults exposed to the September 11, 2001 World Trade Center disaster: results from the World Trade Center registry. Prev Med. 53, 370–376 (2011). 22040652 10.1016/j.ypmed.2011.10.014

[b51] von Känel R. . Evidence for low-grade systemic proinflammatory activity in patients with posttraumatic stress disorder. J Psychiatr Res. 41, 744–752 (2007). 16901505 10.1016/j.jpsychires.2006.06.009

[b52] Shemesh E. . Posttraumatic stress, nonadherence, and adverse outcome in survivors of a myocardial infarction. Psychosom Med. 66, 521–526 (2004). 15272097 10.1097/01.psy.0000126199.05189.86

[b53] Posta L. M., Zoellnerb L. A., Youngstromc E. & Feenya N. C. Understanding the relationship between co-occurring PTSD and MDD: Symptom severity and affect. J Anxiety Disord. 25, 1123–1130 (2011). 21899984 10.1016/j.janxdis.2011.08.003 PMC3196268

[b54] Warren A. M. . Posttraumatic stress disorder following traumatic injury at 6 months: associations with alcohol use and depression. J Trauma Acute Care Surg. 76, 517–522 (2014). 24458060 10.1097/TA.0000000000000110

[b55] Tiihonen Möller A., Bäckström T., Söndergaard H. P. & Helström L. Identifying risk factors for PTSD in women seeking medical help after rape. PLoS ONE. 9, e111136 (2014). 25340763 10.1371/journal.pone.0111136 PMC4207776

[b56] Patel M. B. . Incidence and Risk Factors for ICU-related Posttraumatic Stress Disorder In Veterans and Civilians. Am J Respir Crit Care Med. 193, 1373–81 (2016). 26735627 10.1164/rccm.201506-1158OC PMC4910886

[b57] Auxéméry Y. Posttraumatic stress disorder (PTSD) as a consequence of the interaction between an individual genetic susceptibility, a traumatogenic event and a social context. Encephale. 38, 373–380 (2012). 23062450 10.1016/j.encep.2011.12.003

[b58] Bolduc A. . Identification and referral of patients at risk for Post-traumatic Stress Disorder: A literature review and retrospective analysis. Am Surg. 81, 904–908 (2015). 26350670

[b59] Agnew-Blais J. & Danese A. Childhood maltreatment and unfavourable clinical outcomes in bipolar disorder: a systematic review and meta-analysis. Lancet Psychiatry. 3, 342–9 (2016). 26873185 10.1016/S2215-0366(15)00544-1

[b60] Yehuda R. Post-traumatic stress disorder. N Engl J Med. 346, 108–114 (2002). 11784878 10.1056/NEJMra012941

